# Genomic imbalances in 5918 malignant epithelial tumors: an explorative meta-analysis of chromosomal CGH data

**DOI:** 10.1186/1471-2407-7-226

**Published:** 2007-12-18

**Authors:** Michael Baudis

**Affiliations:** 1 Institute of Molecular Biology, University of Zurich, Winterthurerstrasse 190, CH-8057 Zurich, Germany

## Abstract

**Background:**

Chromosomal abnormalities have been associated with most human malignancies, with gains and losses on some genomic regions associated with particular entities.

**Methods:**

Of the 15429 cases collected for the Progenetix molecular-cytogenetic database, 5918 malignant epithelial neoplasias analyzed by chromosomal Comparative Genomic Hybridization (CGH) were selected for further evaluation. For the 22 clinico-pathological entities with more than 50 cases, summary profiles for genomic imbalances were generated from case specific data and analyzed.

**Results:**

With large variation in overall genomic instability, recurring genomic gains and losses were prominent. Most entities showed frequent gains involving 8q2, while gains on 20q, 1q, 3q, 5p, 7q and 17q were frequent in different entities. Loss "hot spots" included 3p, 4q, 13q, 17p and 18q among others. Related average imbalance patterns were found for clinically distinct entities, e.g. hepatocellular carcinomas (ca.) and ductal breast ca., as well as for histologically related entities (squamous cell ca. of different sites).

**Conclusion:**

Although considerable case-by-case variation of genomic profiles can be found by CGH in epithelial malignancies, a limited set of variously combined chromosomal imbalances may be typical for carcinogenesis. Focus on the respective regions should aid in target gene detection and pathway deduction.

## Background

The analysis of genomic abnormalities in malignant cell clones has been performed for decades. Introduced in the 1960s, the evaluation of stained metaphase preparations from tumor cells [1,2] has been widely employed in basic research as well as in clinical practice. For analysis of complex karyotypes, direct chromosomal inspection was recently enhanced through multicolour in-situ hybridization techniques: combinatorial multi-fluor FISH (M-FISH [3]) and multicolour spectral karyotyping (SKY [4]).

In contrast to traditional or enhanced cytogenetic analysis, molecular-cytogenetic methods rely on tumor DNA as starting material for the exploration of genomic abnormalities. While fluorescent in-situ hybridization (FISH [5,6]) allows for the detection of single or few numerical and structural genomic features in non-dividing cells, Comparative Genomic Hybridization (CGH [7,8]) permits the screening of the whole tumor genome for regional imbalances in the DNA content. Since CGH is based on the hybridization of tumor and reference DNA to standardized normal metaphase spreads, the spatial resolution of this technique is limited to several megabases [9]. Also, the involvement of presumptive target genes can only be inferred from the positional comparison of the genomic ratio profiles to the underlying chromosomal matrix. However, recent molecular-cytogenetic screening techniques (array-or matrix-CGH [10-12]) have the potential for direct identification of oncogenetic target genes.

Based on the comparably easy access to dividing tumor cells, and the early reognition of specific cytogenetic aberrations in some entities [13-15], metaphase analysis has been especially successfull in acute leukemias (review e.g. in [16]) and other hematologic malignancies. This has been reflected in the content of the Mitelman Database of Chromosome Aberrations in Cancer [17], in which hematologic neoplasias account for 73% of the database content (approx. 40,000 cases), while epithelial tumors constitute only 12% (approx. 6,600 cases). In contrast, especially through it's application to the genomic screening of frozen and archival tissue, the potential of CGH for the analysis of solid tumors had been recognized early on [7,18].

Some previous reviews of CGH data have either reported on specific types of aberrations [19,20] or were focused on solid tumors [21] or hematologic malignancies [22]. Struski *et al*. [23] provided a census of 11,984 solid tumors and hematologic malignancies analyzed by CGH. Since in the majority of publications data is presented as summary information (e.g. percentage of frequently involved chromosomal regions), these data reviews were able to develop a granular overview of the major regions frequently imbalanced in different tumor entities. Recently, Myllykangas *et al*. [24] provided an overview of genomic gain/amplification patterns, thereby omitting genomic losses. The study covered a large panel of human neoplasias, based on more than 4500 single cases. The authors were able to show a general relation genomic amplification patterns to general histo-pathological features, but could not prove overall relation of hot-spot sites to some known genetic features.

The current study attempts a descriptive overview of genomic imbalances in epithelial neoplasias, based on published CGH data. Starting point for the data exploration is the Progenetix molecular-cytogenetic database [25-27], which was initiated in December 2000 to collect all published cancer related CGH data and make it available for reference and research purposes. In contrast to other CGH data assembly attempts, but comparable to the Mitelman Database of Chromosome Aberrations in Cancer, complete experimental results are collected on a case-by-case basis. Publications describing (molecular-) cytogenetic analyses of tumor samples and established cell lines are mined for case specific annotation of the experimental results. Focus is the exhaustive collection of chromosomal CGH data, with occasional inclusion of results from Metaphase banding, SKY and M-FISH data, as well as the recent addition of array or matrix CGH [10,28] results.

The detailed discussion of identified or suspected target genes in the different chromosomal regions is not part of this study. However, some examples of genes with oncogene and tumor suppressor function, respectively, will be mentioned.

## Methods

As of 2006-12-04, 15429 cases from 609 publications had been included into the Progenetix database. Of those, 13818 had been analyzed by chromosomal CGH, either alone or in combination with Metaphase analyses techniques (355 cases). Minimum requirement for addition to the database was the availability of case specific data for the complete (molecular-) cytogenetic analysis result and clinical diagnosis. In most cases, locus information was available or could be inferred. Additional data (grading, staging/TNM, age, gender, follow-up parameters) was recorded if accessible. As main disease descriptors, diagnosis and locus information was recoded to ICD-O-3 standard [29], based on the available information.

For the purpose of this article, cases ascribed to epithelial origin (ICD-O 8010/x-8780/x) were selected for further analysis. This base dataset comprised 6899 cases, collected from 254 publications. The oldest included data was from 1994 [30], the latest from 2006 [31]. The largest number of cases was found in a report about skin neoplasias (169 cases; [32]), followed by an extensive comparison of BRCA1 and BRCA2 mutated breast carcinomas (132 cases; [33]). Only 8 articles were single case reports.

To limit the bias in disease entities frequently analyzed in pre-malignant stages, only the 5918 clearly malignant epithelial neoplasias were selected (ICD-O-3 xxxx/2 and xxxx/3). For comparative purposes, common clinico-pathological disease categories were generated through a combination of diagnostic and locus codes (Table 1). Only the 22 entities with more than 50 cases were analyzed separately, while 377 cases were not assigned to one of these entities.

**Table 1:** Distribution of 5918 malignant epithelial tumors by clinico-pathological entities, sorted by frequency of occurrence.

**Diagnosis**	**ICD-O-3 codes^(1)^**	**Locus code(s)**	**No. of cases**
Breast carcinoma	81xx ... 86xx	C50	667
Prostate carcinoma	814x	C61	600
Gastric carcinoma	81xx ... 86xx	C16	529
Ovarian carcinoma	81xx ... 86xx	C56	449
Colorectal adenocarcinoma (CRC)	8140, 8480	C17, C18	430
Hepatocellular adenocarcinoma (HCC)	817x	C22	371
Head-neck squamous cell carcinoma (HNSCC)	8070	C01, C09, C06, C10, C12, C13, C14, C30, C32	339
Thyroid carcinoma	81xx ... 87xx	C73	314
Non-small cell lung carcinoma (NSCLC)	81xx ... 86xx, excl. 8041, 8045, 824x	C34	254
Cervical carcinoma	81xx ... 86xx	C53	226
Esophagus carcinoma (ES)	81xx ... 86xx	C15	209
Renal carcinoma (RCC)	81xx ... 86xx	C64	195
Nasopharynx carcinoma (NPC)	8010, 8070	C11	177
Bladder carcinoma	81xx ... 86xx	C67	169
Neuroendocrine ca. and carcinoid (NE)	824x	(all)	138
Melanocytic (MEL)	872x ... 877x	(all)	99
Pancreas adenocarcinoma (PAC)	814x	C25	88
Cholangio carcinomas	816x	C221, C24	63
Small cell lung carcinoma (SCLC)	8041, 8045	C34	63
Endometrial carcinoma	81xx ... 86xx	C55	56
Vulva carcinoma	81xx ... 86xx	C51	53
Squamous malignancies of the skin (SQS)	807x	C44	52

377 tumors from entities with <50 cases were omitted from this list. (1) ICD codes and locus codes are listed as used for automatic assignment of cases to the disease groups; groups do not necessarily contain cases assigned to the whole range of ICD codes.

During database entry, CGH results were converted to standard "rev ish" ISCN 1995 format. This procedure contained software based syntax errors checking. The corrected "rev ish" data was processed by dedicated software [27] implemented in the Perl scripting language [34] using complex Regular Expression based parsing algorithms. Data matrices were generated, containing the imbalance status ("1" = gain, "-1" = loss, "2" = high level gain) for each of 862 chromosomal bands (UCSC Genome Bioinformatics [35], Golden Path mapping, May 2004 edition), or subsets thereof.

For statistical analysis, software packages available as part of the Bioconductor [36] project for the R programming language [37] were used. Visualization of genomic imbalance profiles was performed by custom Perl routines using the GD.pm interface [38] to the GD graphics library [39]. As a robust measure for genomic instability, the number of chromosomes per case with one or more imbalanced segments was determined.

## Results and Discussion

**Figure 1:**
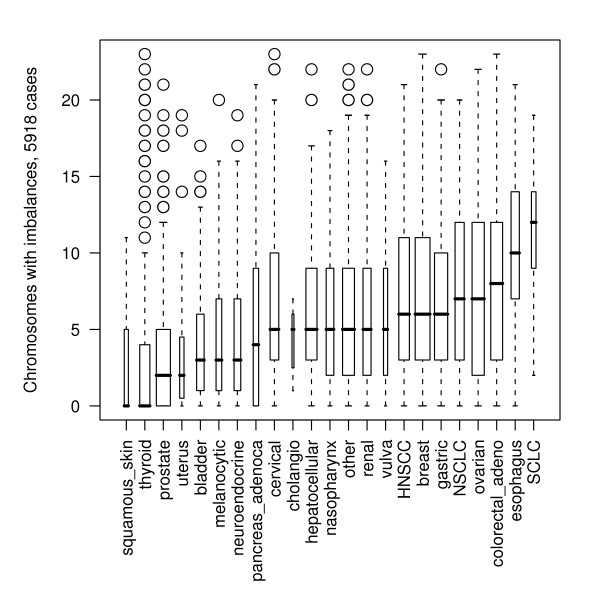
Number of imbalanced chromosomes for different tumor loci as indicator for overall genomic instability, in 5918 malignant epithelial tumors. The box plots indicate the median and distribution of chromosomes in each tumor karyotype, with total or partial genomic imbalances. Only malignant cases (ICD-code ####/2 or ####/3) were analyzed.

In the base data set comprised of 5918 malignant cases analyzed by CGH, breast carcinomas and precursor lesions constituted the largest subgroup, followed by neoplasias of the prostate gland and stomach (Table 1). The median aberration number per case (number of chromosomes with at least one abnormal segment) showed a wide range (Figure 1), from 0 (squamous skin neoplasias, thyroid carcinomas) to 12 (small cell lung carcinomas; SCLC). For some of the entities at the low end of this spectrum, a certain bias through frequent biopsies at early stages can be suspected (e.g. tumors of skin, thyroid, prostate), although pre-maligant tumors had been excluded from the analysis.

### Imbalance hot spots in clinico-pathological entities

**Figure 2:**
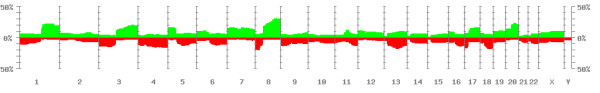
Overall imbalance pattern from all cases. For each chromosomal band (862 bands resolution) the percentage of cases with gains (green, upward) and losses (red, downward) is indicated.

In the overall aberration profile, gains on chromosomal band 8q24 represented the most common imbalance, followed by gains involving regions on 20q, 1q, 3q, 17q, 7(q) and 5p, as well as other changes (Figure 2).

In the following overview, the most frequent regions involved in genomic imbalances are listed, with detailed locus and frequency information for regional hot-spots. Since overall aberration frequencies vary greatly between entities, no absolute cut-off values (e.g. 10% or 20%) were used when selecting these regions. Instead, genomic regions with clear separation from overall background in the imbalance histograms were selected for each entity and sorted in descending frequency of occurrence. Gains and losses were evaluated separately.

For each entity, few outstanding observations are discussed and selected literature is provided. Histologic subtypes are only mentioned in few entities. Because not all involved regions can be discussed here with respect to putative genetic targets, a selection of well characterized oncogenes and tumor suppressor genes with involvement in epithelial neoplasias is provided as part of Table 2. Detailed aberration profiles for each entity can be found in the Additional file 1.

**Table 2:** Occurrence of most common imbalances in different epithelial neoplasias by CGH

**Regional imbalance**	Most frequent*	**Above background**	Putative targets**
**enh(1q) ** occuring in most carcinoma types	**Breast**, ovary, **HCC**, cervix, NPC, MEL, **endometrial**, vulva	Gastric, CRC, HCC, HNSCC, NSCLC, ES, RCC, bladder, PAC, SCLC, SQS	ABL2, ETV3
**dim(3p)**	**HNSCC**, **NSCLC**, cervix, **ES**, **RCC**, NE, MEL (whole chr. 3), **SCLC**, **vulva**, **SQS**	Gastric, PAC	FHIT, MLH1
**enh(3q)** maxima at 3q26q27 (except 3q25 in NPC)	Ovary, **HNSCC**, NSCLC, **cervix**, **ES**, NPC, SCLC, endometrial, **vulva**, SQS	Gastric, RCC, PAC	BCL6, PIK3CA
**dim(4q) ** frequently whole chromosome	Ovary, **HCC**, NSCLC, cervix, ES, bladder, **cholangio**, SCLC	Gastric, CRC, HNSCC, RCC, PAC,	PRDM5
**enh(5p)**	**Thyroid**, **NSCLC**, cervix,	Gastric, ovary, CRC, HCC, HNSCC, ES, bladder, NE, PAC, cholangio, vulva	CDH6, TERT
**dim(5q)**	**Ovary**, NSCLC,	Prostate, gastric, HNSCC, ES, bladder, cholangio,	APC, MCC
**enh(6p)**	HCC, MEL	Ovary, NSCLC, cervix, ES, bladder, cholangio, SCLC, vulva	E2F3, ID4
**dim(6q)** maxima at 6q16q21 or 6q24q27	Prostate, RCC, **MEL**	Ovary, HCC, NSCLC, cervix, bladder, NE, PAC, cholangio, SCLC	CCNC
**enh(7) ** frequently whole 7, mostly max. on 7q	Prostate, thyroid, ES, **RCC**	Gastric, ovary, CRC, HCC, HNSCC, bladder, MEL, PAC (7p>7q), cholangio (7p>7q)	7p: EGFR 7q: ABCB1, MET
**dim(8p)**	Breast, **prostate**, CRC, HCC	Ovary, HNSCC, NSCLC, ES, RCC, bladder, PAC, SCLC, vulva	DLC1, MSR1, N33
**enh(8q)** ubiquitously high (exception NE and thyroid)	Breast, **prostate**, **ovary**, CRC, HCC, HNSCC, NSCLC, ES, RCC, bladder, **MEL**, PAC, **cholangio**, endometrial, vulva	Cervix, NPC, SCLC, SQS	MYC
**dim(9p)** 9p or whole 9	HNSCC, **bladder**, PAC, **SQS**	Gastric, NSCLC, ES, RCC, NPC, MEL, SCLC	ARF, CDKN2A
**enh(11q13) ** frequently distinct (high-level) gain	HNSCC	Breast, gastric, ovary, NSCLC, ES, NPC, bladder, MEL, PAC, cholangio	CCND1, FGF3
**dim(11q23qter)**	NPC, **NE**, vulva	Breast, HNSCC, cervix, ES, MEL, SCLC	ATM (11q22), (LOH11CR2 A, TSG11)
**enh(12p) ** frequently whole 12; slight max. on 12p	**NPC**	Ovary, CRC, HNSCC, NSCLC, ES, RCC, PAC, vulva	12p: CDK2, CDK4, GLI, KRAS 12q: MDM2
**dim(13q)** mostly 13q14q21	Prostate, HCC, **thyroid**, bladder, NE, PAC, SCLC, endometrial	Breast, gastric, ovary, HNSCC, NSCLC, cervix, ES, RCC, NPC (max. at 13q31), MEL, CRC, vulva	BRCA2, RB1, STARD13
**dim(16q)**	**Breast**, **NPC**	Prostate, gastric, HCC, SCLC	CDH1, ATBF1
**dim(17p)**	Breast, **gastric**, CRC, cholangio, SCLC	Ovary, HCC, NSCLC, cervix, ES, RCC, NPC, bladder, PAC, SQS	TP53
**enh(17q)**	Breast, gastric, bladder, NE, PAC, cholangio, SCLC, SQS	HCC, HNSCC, NSCLC, cervix, ES, renal, NPC	ERBB2
**dim(18q)**	Gastric, ovary, **CRC**, HNSCC, **PAC**, SQS	HCC, NSCLC, cervix, renal, bladder, cholangio	DCC, SMAD4
**enh(19q)**	**NE**, **SCLC**	Breast, CRC, PAC, vulva	AKT2, BAX
**enh(20q)**	**Gastric**, **CRC**, thyroid, **bladder**, NE, **PAC**, cholangio	Breast, HCC, cervix, ES, renal, MEL, SCLC, vulva	STK15/AuroraA

Please refer to table 1 for abbreviations. * "most frequent" lists entities in which the aberration belongs to the 3 most frequent imbalances of the specified quality (enh = gain, dim = loss; bold if most frequent change in entity). Entities are sorted according to the total number of included cases. Chromosomal regions 1p, 21, 22, X and Y were omitted due to differences in reporting (e.g. exclusion of regions prone to errors in chromosomal CGH analysis). ** Listed are some examples of genes with oncogene (for gain regions) or tumor suppressor (for loss regions) function. However, a large number of possible target genes as well as structural features may exist for each region.

#### Breast carcinoma (667 cases)

Gains: 1q31 (50.8%), 8q23 (47.3%), 17q24 (31,2%), 20q (30.9%), 16p, 11q13, 19, 3q

Losses: 16q (30%), 8p23 (26.1%), 17p13 (23.5%), 11q23 (23.1%), 13q21 (22.8%)

In breast carcinomas, gains on 1q and losses on 16q constitute the most frequent copy number changes of each quality. In the literature, a cytogenetic subtype with combination of these imbalances and few other genomic changes has been associated with histology of well differentiated DCIS [40] and a favorable prognosis [41]. Interestingly, gains on 11q13 and 12q24 were associated with higher metastasis-free survival in another study [42], which also associated multiple imbalances with a bad prognosis.

Although ERBB2 has been a known and clinically relevant [43] amplification target on 17q, distinct amplicons mapping telomeric to the ERBB2 locus have been described [44].

#### Prostate carcinoma (600 cases)

Gains: 8q24 (23%), 7, X

Losses: 8p21 (32.3%), 13q21 (26.5%), 6q16 (18.5%), 16q(23), 5q(21)

In contrast to other adenocarcinomas, and accounting for the overall low copy number variations, prostate ca. usually lack the 1q gains frequent in most carcinoma entities. While Mattfeldt *et al*. [45] showed a correlation of 8p loss to higher tumor stage, the negative prognostic impact of gains of 8q and chromosome 7 in was reported from bioptic samples [46] and early tumors [47]. A meta analysis of published copy number data recently was provided by Sun *et al*. [48].

#### Gastric carcinoma (529 cases)

Gains: 20q12 (36.1%), 8q23 (31.7%), 17q21 (21.2%), 7 (up to 20.8%), 13q22q31, 1q, 3q, 5p, 11q13, X

Losses: 17p13 (24.4%), 19p (18%), 18q (16.6%), 1p, 3p, 4, 5q, 9p, 12q, 16

For most chromosomes, an overall background of 4–10% of gains as well as losses can be found. In gastric carcinomas as well as in some other entities, 8q gains have shown their maximum at 8q23, proximal to the c-myc locus [49], implying a hitherto unidentified target.

#### Ovarian carcinoma (449 cases)

Gains: 8q24 (43.7%), 3q26 (38.1%), 1q32 (25.3%), 12p12 (19.2%), 2, 5p, 6p, 7q, 11(q13)

Losses: 5q14 (25.2%), 4q (26.3%), 18q21 (19.8%), 17p (19.6%), 8p (19.4%), 6q, 9q, 13q, 16q, X

Gains on 12p have been discussed as early event in ovarian carcinomas with frequent occurrence in borderline tumors [50], and several other changes have been linked to advanced carcinomas [51]. Overall, advanced stage tumors showed a higher grade of chromosomal instability.

#### Colorectal adenocarcinoma (430 cases)

Gains: 20q13 (53%), 13q (38.6%), 8q24 (37.2%), 7(p) (35.6%), X(q21), 1q, 5p, 12(p), 19

Losses: 18q(22) (47.4%), 8p(22) (37.9%), 17p12 (27%), 4 (up to 23.7%), 14, 15, 22

In colorectal carcinoma, gains on 8q23q24 [52] have been associated with lymph-node positivity. In a recent study, gains on 20q as well as KRAS mutations could bee shown to precede aneuploidy [53]. An overview is provided in [54].

#### Hepatocellular adenocarcinoma (HCC; 371 cases)

Gains: 1q23q31 (46.6%), 8q24 (44.8%), 6p21 (22.4%), 17q (21.8%), 5, 7, 20

Losses: 4q (up to 31.3%), 8p(21) (31.3%), 13q21 (28.3%), 16q(21) (25.9%), 17p13 (25.3%), 1p, 6q, 14, 18

Losses of 8p have been shown to distinguish HCC from other liver malignancies [55]. Also, losses on 4q and 13q were associated with poor differentiation [56]. An overview of genomic changes in HCC with discussion of putative target genes is provided in [57].

#### Head-neck squamous cell carcinoma, excluding nasopharyngeal ca. (HNSCC; 339 cases)

Gains: 3q26 (59.2%), 8q24 (40.8%), 11q13 (31.9%, many specific high-level), 5p (26.5%), Xq, 1q, 7q(21), 12p, 17

Losses: 3p (30.1%), 18q(22) (22.4%), 9p (22.4%), 11q24 (19.2%), 4, 5q, 8p, 13

Genomic imbalances in HNSCC are in line with other squamous cell carcinomas (-3p, +3q, +5p). The high rate of amplifications at 11p13 has been shown to involve the cyclin D1 (CCND1) locus and to be accompanied by high expression of the gene [58].

#### Thyroid carcinoma (314 cases)

Gains: 5(p), 7, 20(q)

Losses: 13q, 22, 1p

Although a number of highly aberrant cases was included (see outliers in box plot, Figure 1), thyroid carcinomas, including all variants, showed an overall the lowest level of chromosomal imbalances. The subset included a large study in post-Chernobyl tumors in children, for which imbalances could be found only in 30% of tumors [59]. Interestingly, anaplastic carcinomas had a low median number but high variability. Although not mapping to regions with frequent genomic gains in the the included cases, previous reports have shown e.g. amplification of PRKCE on 2p21 [60] and FGF3 [61].

#### Non-small cell lung carcinomas (NSCLC; 314 cases)

Gains: 5p (52.8%), 3q26 (39.4%), 8q24 (35.4%), 1q(21) (28.4%), 3, 6p, 11q13, 12p, 17q, 18p

Losses: 3p (31.9%), 4(q) (26.4%), 5q (26%), 13q21 (25.6%), 8p(21) (24%), 9 (up to 20.5%), 17p (16.5%), 1p, 6q, 10, 18q

NSCLC is a histologically heterogeneous group, consisting of squamous (SCC) and non-squamous (NSCC) cases. Imbalances have been shown to be partially shared between the groups, with differences in the frequency of some regional involvements [62].

#### Cervix carcinoma (226 cases)

Gains: 3q26qter (53.6%), 1q (up to 28.3%), 5p (27.4%), 8q24 (20.3%), 6p, 9(q), 17q, 20q, X

Losses: 2q36 (30.1%), 3p (up to 25.2%), 4 (up to 23.5%), 11q23q24 (23%), 6q, 13q, 17p, 18

The number of chromosomal changes may be influenced by occurrence and type of HPV infection. However, in a large study no correlation between single imbalances and clinical parameters could be identified [63]. Losses of the telomeric region of 2q were frequent and rarely found in other entities.

#### Esophagus carcinoma (209 cases)

Gains: 3q(26) (53.2%), 8q24 (49.8%), 7p (34%), 20q13 (32.6), 1q (29.7%), 7q21 (29.7%), 5p (28.7%), Xq (23.4%), 6p12 (22.9%), 12p (22.5%), 11q13 (22.1%), 17q21 (21.6%), 2(q), 9q

Losses: 3p (up to 37.3%), 18q (37.3%), 4 (up to 33%), 5q(21) (29.2%), 9p (26.3%), 8p (23.4%), 13q (up to 23%), 1p (22.5%), 19q (2%), 17p (20.1%), 10, 11, Xp

Esophagus carcinomas showed an overall high "background" of gains and losses, with the 2nd highest number of imbalances per case. This group consists of cases with squamous cell as well as adenocarcinoma or intestinal adeno-ca. histology. Besides specific de-regulation of oncogenes, an effect of the massive genomic changes on chromatin structure has been discussed previously [64].

#### Renal carcinoma (RCC, 195 cases)

Gains: 7 (up to 33.4%), 5q(31) (32.8%), 8q23q24 (19.5%), 20 (up to 18.5%), 17q (up to 17.4%), 1qter (13.1%), 3q, 12, 16

Losses: 3p (up to 43.1%), 14q (up to 26.2%), 6q (21%), 1p (up to 20%), 9 (20%), 8p (19.5%), 13q (19.5%), 17p (19%), 18q (18%), 2, 4, 10

Gains of 5q are a rare occurrence in other carcinomas, and have been identified in papillary as well as non-papillary cases [65]. An overview of accumulated karyotype data was given in 2004 [66], proposing different cytogenetic pathways and associating papillary RCC with a hyperdiploid karyotype pattern.

#### Nasopharynx carcinoma (NPC; 177 cases)

Gains: 12p (up to 33.9%), 1q (24.3%), 3q (22%), 8q22q23 (19.5%), 18p (17%), 11q13 (12.4%), 2(q), 4(q), 6(q), 17q

Losses: 16q (29.9%), 14q24 (27.7%), 11q23 (24.3%), 1pter (23.7%; difficult region), 9, 13q31, 17p, 19p

In contrast to some other carcinoma entities, the maximum of gains mapped clearly proximal to the *c-myc *region. A meta-analysis of CGH data in NPC was recently provided by Li *et al*. [67]. In concordance with the low number of NPC cases with 8p deletion, the oncogenetically relevant DLC1 on 8p22 has been reported to be inactivated by methylation rather than copy number change [68].

#### Bladder carcinoma (169 cases)

Gains: 20q (21.3%), 8q (20.7%), 17q (20.7%), 11q13 (19.5%), 1q21 (17.8%), 5p, 6p, 7, 10p

Losses: 9 (26%), 13q21 (17.8%), 4q (up to 14.2%), 11p (14.2%), 5q (14.6%), 2q32, 8p, 6q, 18q, X, 17p

While some of the imbalances (e.g. 8q, 17q and 20q gain, 11q13 gain/amplification) were frequent in other entities, losses on chromosome 9 were exceptionally high in transitional cell carcinoma (115 cases). In contrast to other squamous cell carcinomas, gains on 3q were rare (3 of 40 bladder SCC). In one study included in the data, some differences between histological and etiological subtypes of bladder carcinomas were presented [69].

#### Neuroendocrine carcinoma and carcinoid (138 cases)

Gains: 19 (26.1%), 20 (up to 18.1%), 17q (up to 17.4%), 5p (15.9%)

Losses: 11q22q25 (20.3%), 13q21 (18.1%), 3p (up to 15.5%), 6q22 (13%), 10q25q26 (13%)

Possibly due to the heterogeneity of this group, a diffuse background of whole chromosome gains was observed. The genetics of neuroendocrine tumors was recently reviewed with consideration for inherited syndromes as well as molecular cytogenetic results [70].

#### Malignant melanocytic neoplasias (99 cases)

Gains: 8q(21) (36.3%), 6p (32.3%), 1q (22%), 7, 20q, 11q13

Losses: 6q(24) (29.3%), 10 (q22) (22.2%), 3 (up to 21.2%), 9p (21.2%), 13q, 1p, 11q23q24

In contrast to most epithelial malignancies, gains on 6p can be found in a large proportion of malignant melanomas. Above background losses on 10q are found in few other entities, too. Similar to other entities gains on 8q, 1q, 20q and chromosome 7 can be observed. A comprehensive overview about molecular-cytogenetic techniques in the analysis of melanocytic lesions was given by Bauer and Bastian [71].

#### Pancreas adenocarinoma (88 cases)

Gains: 20q (up to 30.7%), 8q(23) (26.2%), 17q (up to 21.6%), 12p11 (19.3%), 7p (18.2%), 3q (up to 17.1%), 5p, 11(q13), 14, 15qter, 16, 19q

Losses: 18q (35.2%), 9p(23) (29.6%), 13q (18.2%), 3p (up to 17.1%), 6q(21) (17.1%), 8p (17.1%), 17p13 (15.9%), 4, 15q, 10q, 12q

This subset only includes pancreas adenoocarcinomas, omitting endocrine tumors.

High level copy number amplifications have been shown e.g. for ERBB2 (17q12) and EGFR (7p12). Interestingly, the maximum for gains on 8q (8q22) is proximal to the *c-myc *region. This fact was also mentioned in a recent overview of genomic screening results in pancreas ca. [72].

#### Cholangio-carcinomas (intra- and extrahepatic; 63 cases)

Gains: 8q22 (42.9%), 17q (up to 39.7%), 20q (34.9%), 11q13 (23.8%), 15q (up to 23.8%), 3q(26) (20.7%), 7p (up to 20.7%), 13q (up to 20.7%), 5p (up to 19.1%), 1q, 6p, 12q23q24

Losses: 1p34p36 (up to 22.2%), 4q (up to 20.6%), 17p (19.1%), 5q(14), 6q, 13q, 18qter, X

Gains on 15q were reported in up to 36% (in a study of Korean intrahepatic cholangio-ca. [73]), but rarely seen in other entities.

#### Small-cell lung carcinoma (SCLC; 63 cases)

Gains: 19 (up to 44.4%), 3q (up to 41.3%), 17q23 (41.3%), 1p31p34 (38.2%), 8q(23) (36.5%), 20q (up to 30.2%), 14q31q32 (26%), 1q (up to 25.4%), 9q31q34, 6p, 13q32

Losses: 3p (84.1%), 4 (up to 74.1% on 4qter), 13q(14) (69.8%), 10(q) (41.3%), 17p13 (33.3%), 2(q23q24) (31.8%), 8p (25.4%), 9, 11, 15, 6q, 16q

SCLC had the highest number of chromosomal imbalances (median 12 involved chromosomes per case). Also, some of the imbalances constituted the overall highest fractional aberrations in all datasets (e.g. >80% losses on 3p), as well as very frequent losses on 4q, 13q and 10q. Although frequent gains on 7p had been reported from array CGH of SCLC cell lines [74], this region was rarely involved in the mostly clinical specimen analyzed by chromosomal CGH.

#### Endometrial carcinoma (56 cases)

Gains: 1q (50%), 8q(22) (39.3%), 3q26 (14.3%), 10 (up to 14.3%)

Losses: 10q (12.5%), 13q (10.7%), 14q, 9q

As in e.g. nasopharynx ca., SCLC and pancreas ca., the maximum of 8q gains was proximal of 8q24. Endometrial ca. show an exceptional high rate of gains on 1q, which were also the most frequent change in a series of 98 cases not available for inclusion here [75]. Interestingly, chromosome 10 was also frequently involved (both gains and losses).

#### Carcinomas of the vulva (53 cases)

Gains: 3q (45.7%), 8q(23) (34%), 1 (up to 28.3%), 5p(15) (26.4%), 20q (24.5%), 9q (20.8%), 6, 7, 12, 14, 19, X

Losses: 3p (22.6%), 4p (22.6%), 11q23q25 (20.8%), 13q14 (13.2%), 8p, 10, X

Vulvar carcinomas showed an imbalance pattern characteristic for squamous cell neoplasias, with a high rate of gains on 8q23 and especially 3q. As in cervix ca., the most frequent changes could not be related to HPV status [76].

#### Squamous cell carcinomas of the skin (52 cases)

Gains: 3q (11.5%), 17q (11.5%), Xq (9.6%), 1q, 8q, 14q

Lossses: 9p21 (19.2%), 3p (13.5%), 18q (13.5%), 17p (9.6%)

While containing many cases of borderline malignant behaviour, squamous skin neoplasias had the overall lowest aberration frequencies (median 0). This group did not include the pre-malignant keratoacanthomas, for which a frequent gain of 11q and cyclin D1 overexpression had been shown [77].

### Disease-specific Involvement of most frequently aberrant chromosomes and relationship of overall aberration profiles

**Figure 3:**
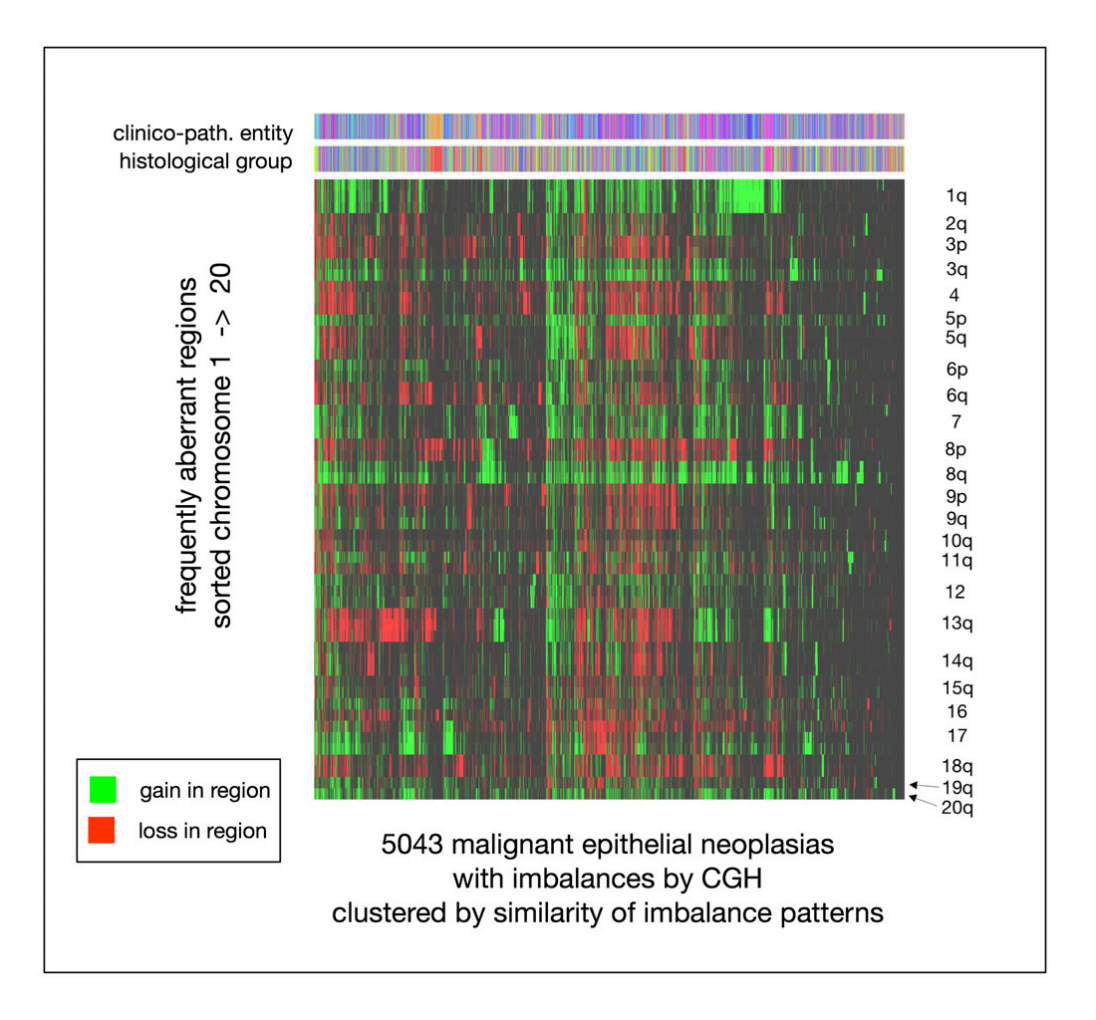
Clustering of 5043 malignant epithelial neoplasias by the pattern of gains and losses, using regions previously defined as highly aberrant in one or several entities (ref. tables 2 + 3). For each case (x-axis), gains (green) and losses (red) are indicated for the corresponding chromosomal regions (55 selected bands; y-axis). The color bar codes on top indicate the cases' assignments to the different clinico-pathological entities and histological groups (color code is provided in the additional file 1).

Several chromosomal hot spot regions showed a pronounced disease related variation. The most prominent regions scoring high in specific entities are listed in Table 2. To reduce sampling bias (e.g. entity specific inclusion of a high proportion of early stage cases) top-scoring aberrations were defined as the most frequent gains or losses in each entity. Some additional imbalances which occured only in few entities at high levels are listed in Table 3. In an attempt to identify the relation of single case aberration profiles, a cluster analysis of all 5043 informative cases was performed. For that purpose, a band specific matrix with 86 bands resolution was generated, of which the 55 intervals most frequently involved in imbalances were selected (top-scorers in one or more entities, ref. Tables 2 and 3; e.g. "8q2"). Hierarchical cluster analysis revealed the complexity of the case-specific aberration patterns, and was able to visualize concordance of imbalance patterns and disease categories in small groups of cases (Figure 3).

**Table 3:** Selected imbalances with high penetrance limited to one or few entities

**Imbalance**	**Occurrence**	**Comment**
**dim(2q22q31)**	SCLC (up to 31.8%)	Profile appears region-specific; however, SCLC cases have very rich imbalance pattern with more frequent changes
**dim(2q33q37)**	**Cervix **(up to 30.1%)	Specific terminal 2q deletions are else only found in low frequency in NSCLC and bladder lesions
**enh(5q21q35)**	RCC (up to 32.8%)	Specific regional 5q gains are near exclusive for renal carcinomas, and are here part of whole chromosomal changes (20%) or limited to the region
**dim(10q)**	SCLC (up to 41.3%), melanocytic, **endometrial**	Rare as specific changes above "background"
**enh(13q)**	CRC (up to 38.6%), gastric	13q gains are rare except in CRC, esp. compared to the frequent losses in the region

**Figure 4:**
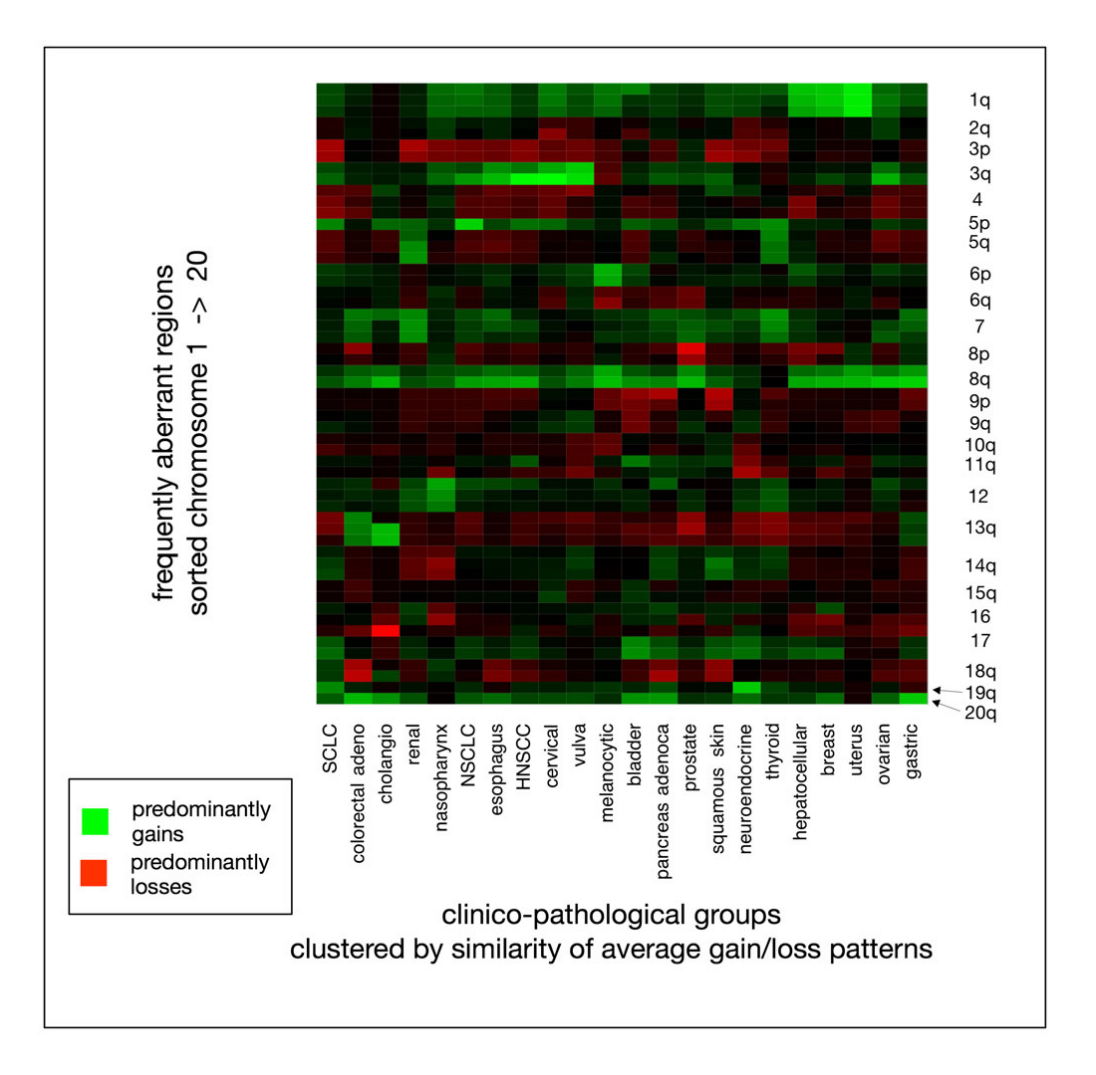
Clustering of carcinoma entities by their overall imbalance pattern. For each of the selected chromosomal regions, aberrations were summarized (percent gain – percent loss). After normalization of all regions over the respective entity, the color intensities represent the relative contribution of regional gains and losses to the overall aberration patterns.

To reduce the complexity of the data and simplify the detection of similarities in the aberration patterns of different entities, region specific aberration frequencies of all entities were clustered. For each of the 55 selected intervals, the sum of gains – losses was calculated, resulting in a 22 × 55 matrix (entities × intervals). During hierarchical cluster analysis, values were normalized over each entity's intervals, to account for differences in genomic complexity. Figure 4 depicts the result of the cluster analysis, in which overall similarities and differences in the different entities become apparent.

**Figure 5:**
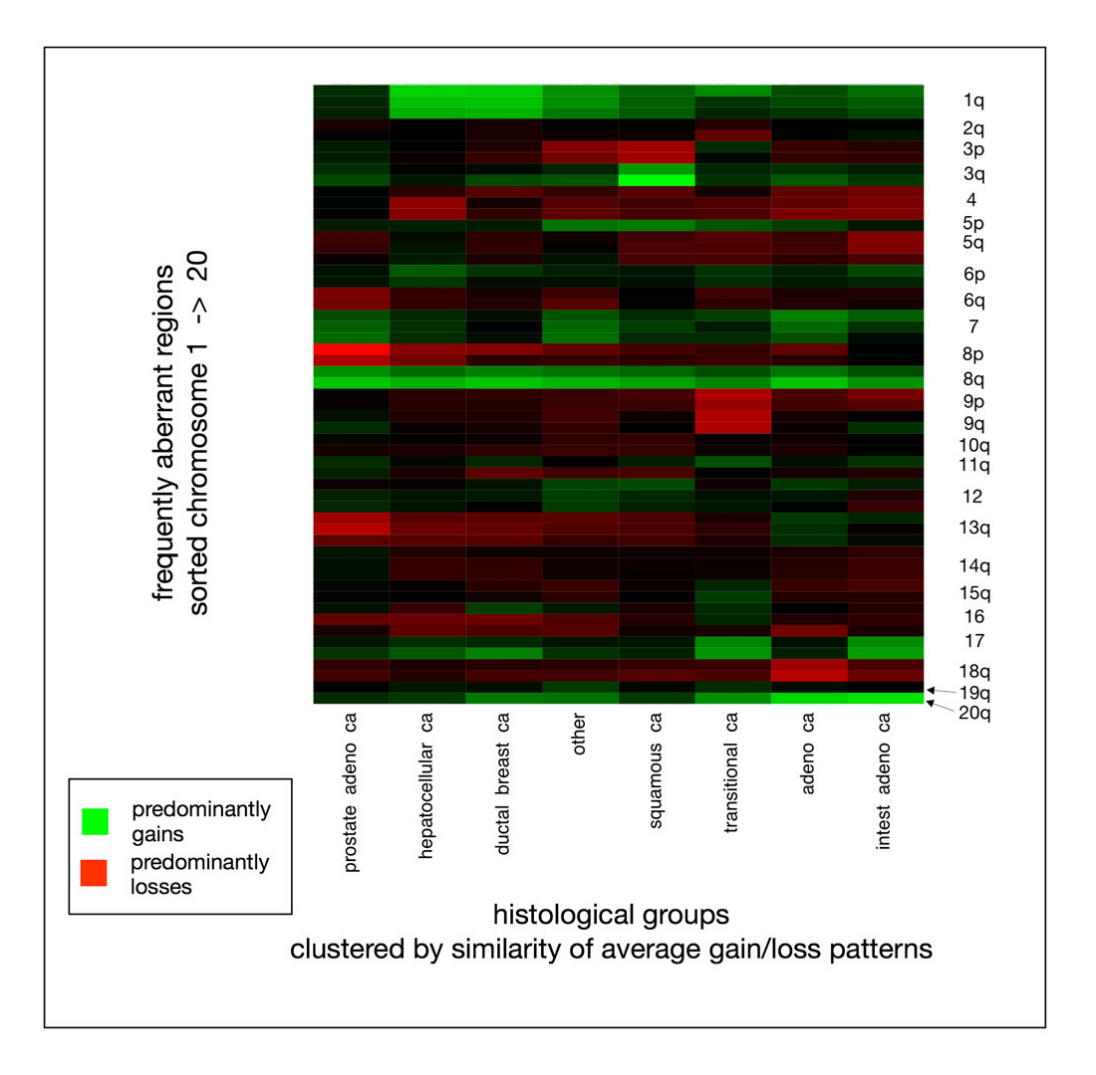
Clustering of different histologies in carcinomas by their overall imbalance pattern. Here, the most frequent histological types were automatically grouped for their overall imbalance profiles. Since considerable differences had been found for adenocarcinoma cases from the prostate (most notably lack of 1q gains), this group was separeted from the overall adenocarcinoma group.

Differences in aberration profiles in some instances will reflect the general histological type, which is substantiated by the close relation of overall profiles e.g. from clinico-pathological groups containing squamous cell carcinoma cases (group NSCLC/esophagus/HNSCC/cervical/vulva in Figure 4). To evaluate this effect, the most frequent histopathological entities (adenoca., squamous cell ca., hepatocellular ca., ductal breast ca., transitional cell ca., intestinal adenoca.) were selected. Additionally, adenocarcinomas of the prostate were put in a separate group, due to the previous observation of specificities in the imbalance profile (e.g. overall lack of 1q gains). Clustering was performed analogous to the method described above. Figure 5 visualizes the clustering of those histopathological entities.

In comparing the most frequent imbalances for their occurrence in different entities, some general observations stood out. Gains involving the terminal part of 8q were ubiquitously found, and often belonged to the most frequent imbalances in the respective entities. Hovever, they were rarely detected in neuroendocrine carcinomas and thyroid neoplasias. Interestingly, in some entities the maximum of detected abnormalities mapped proximal of 8q24 (e.g. 8q21 in melanocytic NPL and 8q22 in NPC and cholangio ca.). How much this reflects a true difference in regional involvement, and therefore may point to differential target gene involvement has to be left open here.

Gains on 1q with maxima on 1q2 were also frequent, and were the most frequent changes in breast, hepatocellular and endometrial carcinomas. However, 1q gains were comparatively infrequent in carcinomas of the prostate and bile ducts as well as in renal and colorectal ca. Overall gains on 3q26q27 combined with losses on 3p were characteristic for squamous cell carcinomas and SCLC, with 3q gains also being frequent in ovarial ca. and other entities.

While 5p gains were found in various entities, gains on 5q were frequent only in ca. of thyroid and kidney, as were gains on chromosome 12. Overall, 13q losses could be detected in most entities. In contrast, 13q gains were predominant in cholangio ca. and also frequent in colorectal and gastric carcinomas.

**Figure 6:**
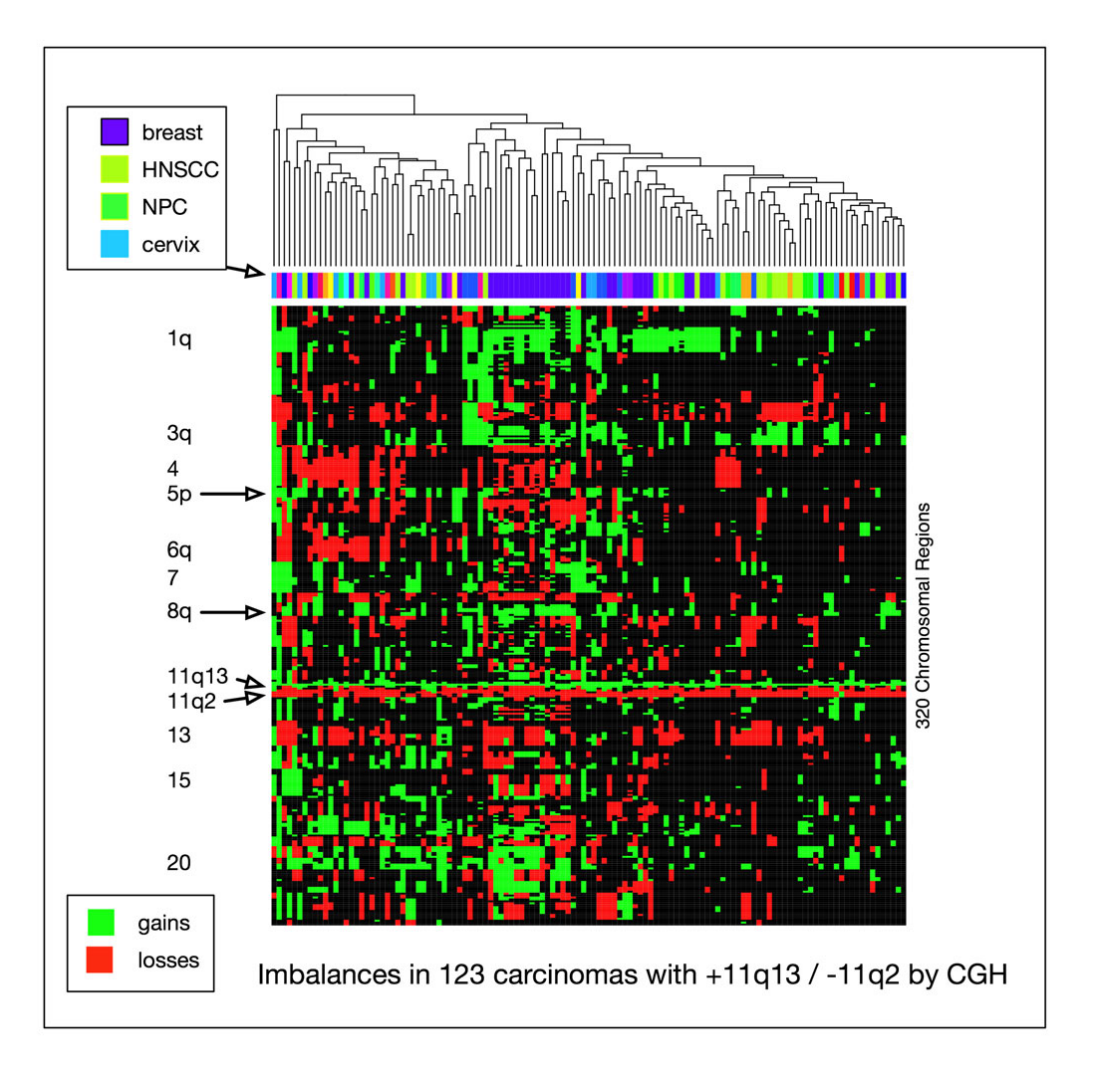
Visualization of single case aberration patterns in 123 carcinomas with gain on 11q13 and concomitant proximal loss. Cases are clustered according to their imbalance patterns (gain/loss status, 320 bands resolution).

Region specific gains involving 11q13 were found in a number of entities (bladder ca., HNSCC, pancreas, prostate, skin, ovary, gastric, NSCLC). Interestingly, of the overall 692 cases with gain on 11q13, 18% had a loss of the telomer of 11q by CGH (Figure 6). Since a strong selective pressure for such a switch in aberration quality in linked regions can be suspected, this observation may point towards targeting of both genes with oncogene and tumor suppressor functions on 11q in these tumors.

When comparing overall aberration patterns, entities containing squamous cell carcinoma cases (HNSCC, NSCLC, cervix and vulva ca., esophagus ca.) appeared related, with a common pattern offrequent losses on 3p, gains on 3q, 8q and varying gains on 1q and 5p. NPC showed a slightly diverging pattern, with predominant 12p gains and 14q losses in addition to slightly lower levels of those imbalances.

Interestingly, in both unselected renal carcinomas as thyroid neoplasias, the average imbalance profiles included frequent gains on chromosomes 5, 7 and 12, while lacking the dominance of gains 1q and 8q found in most other entities.

Apart from these observations, one should refer to the accompanying figures for a general comparison of patterns in different clinico-pathological groups. Also, the use of the Progenetix website tools is encouraged.

## Conclusion

This study attempted an overview of chromosomal imbalance profiles in epithelial neoplasias, based on a large collection of case-specific chromosomal imbalance data from published chromosomal CGH experiments. The most striking observations were the high frequency of a limited set of changes (+1q, +3q, +7, +8q, -13q, -17p, -18q, +20q ...), of which different subsets occurred in most entities. The iterative involvement of certain genomic regions in either gains or losses is a strong argument for the non-random incorporation of these changes into the tumor cell genomes, with common as well as disease specific changes becoming apparent. One may speculate that the recurring combination of a limited set of imbalance hot-spots is concordant with the multistep process of cancer development [78], and that these regions point towards preferential oncogenetic targets. For a detailed discussion of the region-specific changes and patterns related to single disease entities, only examples could be given in the context of this article. The existing literature should be considered for additional information.

This descriptive analysis of local genomic imbalance frequencies cannot be able to address some important questions, e.g. regarding the biological significance of the observed changes. Although certain genomic copy number amplifications and segmental deletions have been shown to involve oncogenetically relevant genes in various malignancies (see Table 2 and for reference e.g. [79-82]), some of the recurring genomic imbalances might be as well pure epi-phenomena of defects in the molecular maintenance of the genome. In respect to the limited spatial resolution and phenomenological nature of the data, the discussion and validation of specific target genes should be left to meticulously crafted molecular-biological experiments.

Cluster analysis of case specific chromosomal CGH data has been used previously to identify subsets of cases in single clinico-pathological entities [45,83,84]. Current methodology appears more suited for limited data sets, while new algorithms have to be developed for automatic subgroup detection from large, heterogeneous series of CGH data [85,86]. This is especially challenging regarding the variant background of chromosomal copy number changes found in certain tumor types, which may be based on aberrant expression of regulatory genes [41]. Since genomic copy number changes constitute only one of many mechanisms leading to aberrant gene regulation, the deduction of oncogenetic pathways from CGH data will remain a challenging project. However, the combination of existing large-scale data collections and current high resolution screening techniques should provide additional pieces for these puzzles. For that purpose, the Progenetix data collection is open for inclusion into data mining projects.

## Competing interests

The author(s) declare that they have no competing interests.

## Authors' contributions

MB designed the study, assembled the data collection, performed the data analysis and wrote the manuscript.

## Pre-publication history

The pre-publication history for this paper can be accessed here:



## Supplementary Material

Additional file 1 Supplementary figures. The file contains imbalance histograms for each of the different clinico-pathological entities and additional box plots depicting the number of aberrant chromosomes per entity and histological subset. Click here for file
