# Supplementary material for: Genomic imbalances in 5918 malignant epithelial tumors: an explorative meta-analysis of chromosomal CGH data
Source: BMC Cancer. 2007 Dec 18;7:226. doi: 10.1186/1471-2407-7-226 (PMC2225423; doi:10.1186/1471-2407-7-226)
Supplement: Additional file 1 — Supplementary figures. The file contains imbalance histograms for each of the different clinico-pathological entities and additional box plots depicting the number of aberrant chromosomes per entity and histological subset. [file 1471-2407-7-226-S1.pdf]

Genomic imbalances in 5918 malignant epithelial tumors: An explorative meta-analysis of chromosomal CGH data.

Supplementary Figures

Michael Baudis

Institute of Molecular Biology, University of Zurich, Winterthurerstrasse 190, CH-8057 Zurich

Email: mbaudis@gmail.com

Overall imbalance profile

As in figure 2 in the article, imbalance profiles (histograms) show the region specific frequency of genomic gains (upwards, green) and losses (downwards, red).

5918 malignant epithelial neoplasias analyzed by CGH

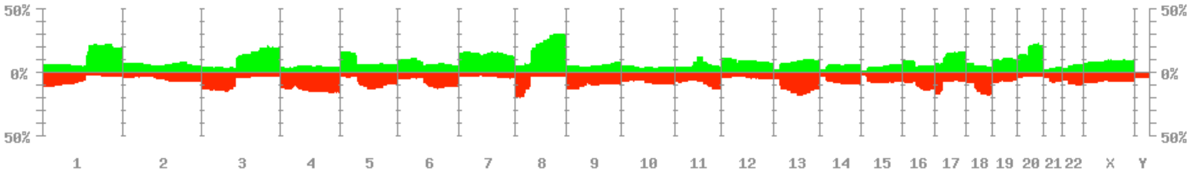

Disease-specific imbalance profiles

Breast carcinoma (667 cases)

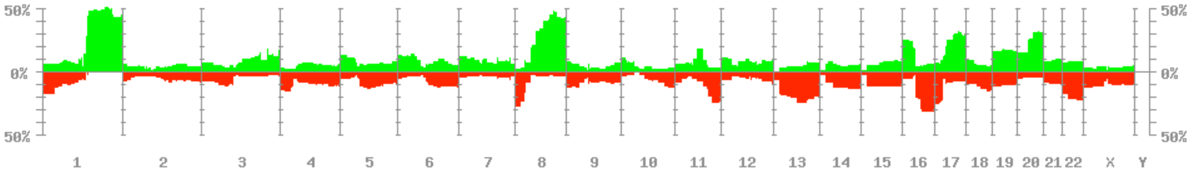

Prostate carcinoma (600 cases)

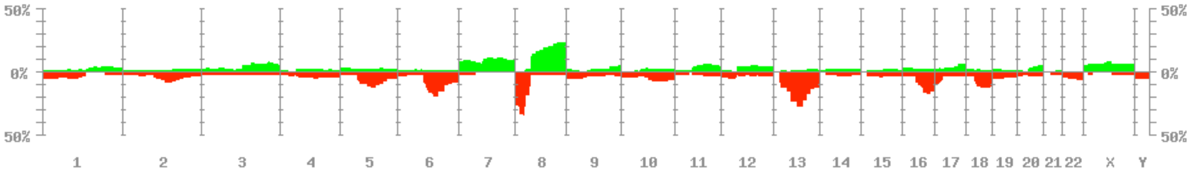

Gastric carcinoma (529 cases)

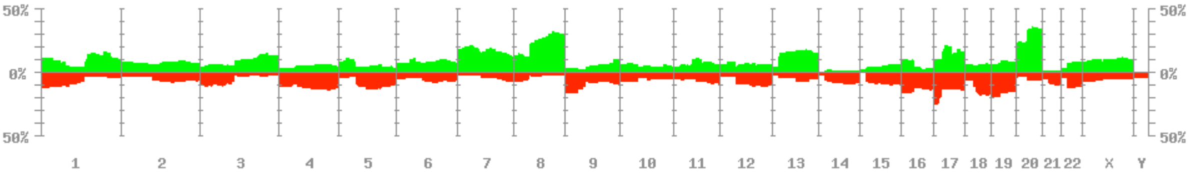

Ovarian carcinoma (449 cases)

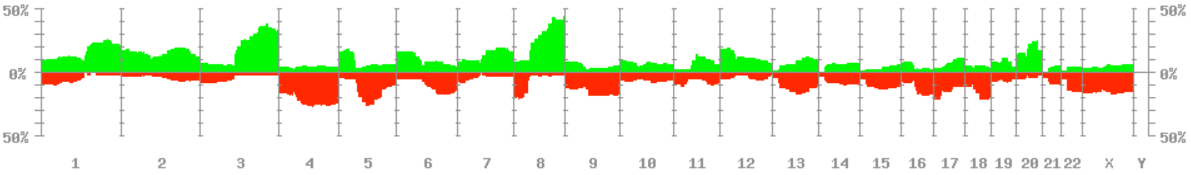

Colorectal adenocarcinoma (430 cases)

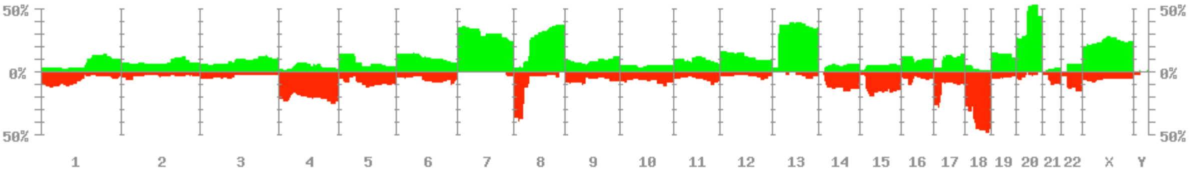

Hepatocellular adenocarcinoma (371 cases)

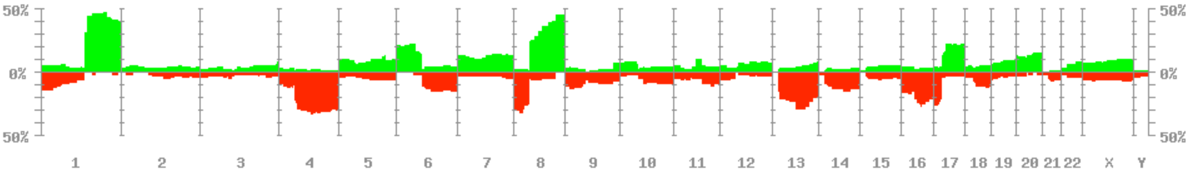

Head-neck squamous cell carcinoma, excluding NPC (339 cases)

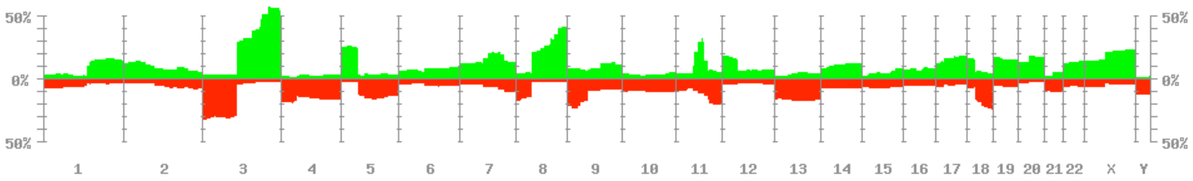

Thyroid carcinoma (314 cases)

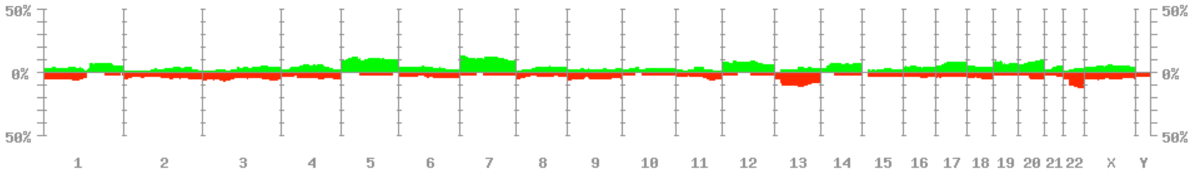

Non-small cell lung carcinomas (NSCLC, 314 cases)

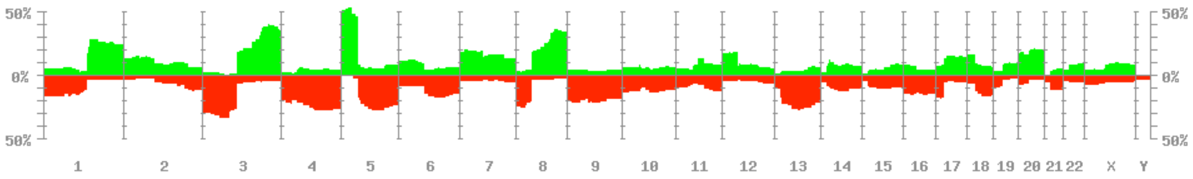

Cervix carcinoma (226 cases)

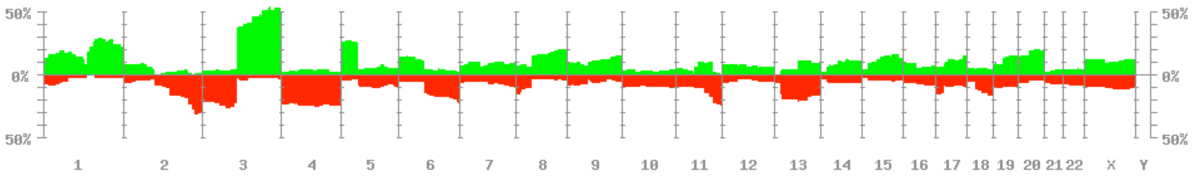

Esophagus carcinoma (209 cases)

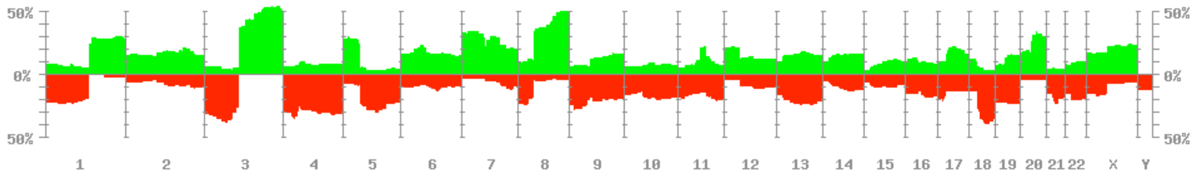

Renal carcinoma (RCC, 195 cases)

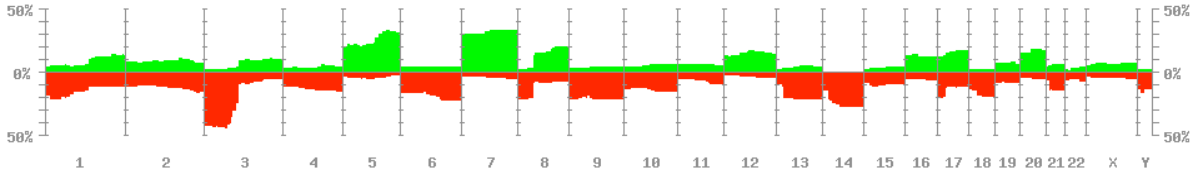

Nasopharynx carcinoma (NPC, 177 cases)

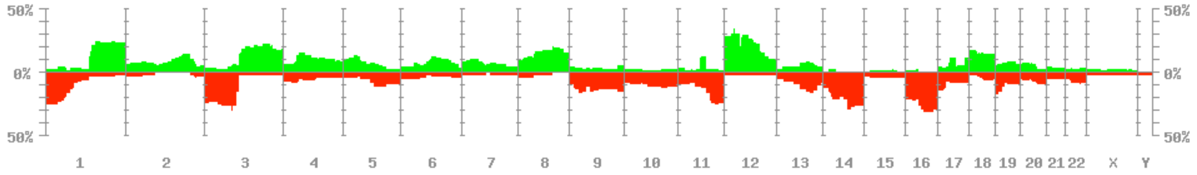

Bladder carcinoma (169 cases)

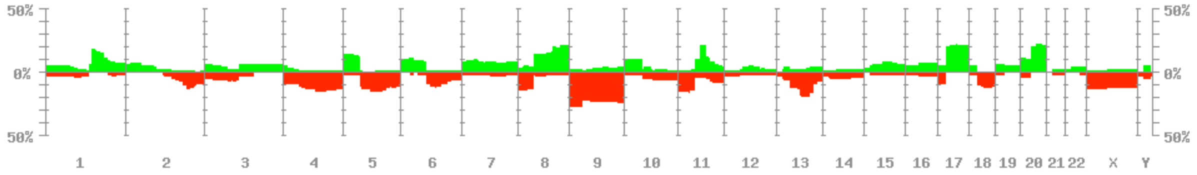

Neuroendocrine carcinoma and carcinoid (138 cases)

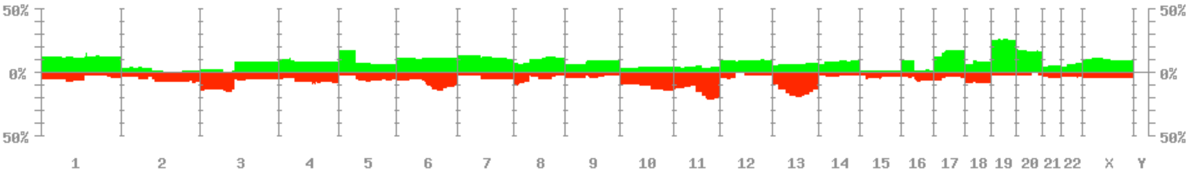

Malignant melanocytic neoplasias (99 cases)

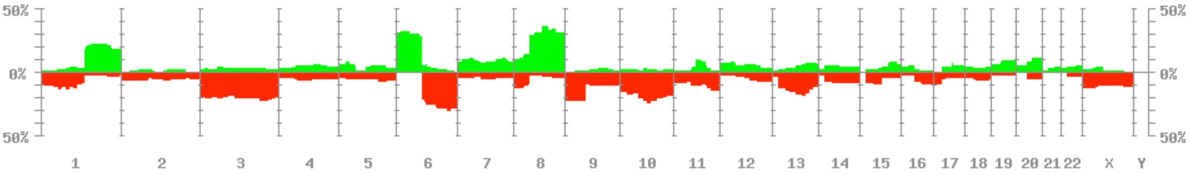

Pancreas adenocarinoma (88 cases)

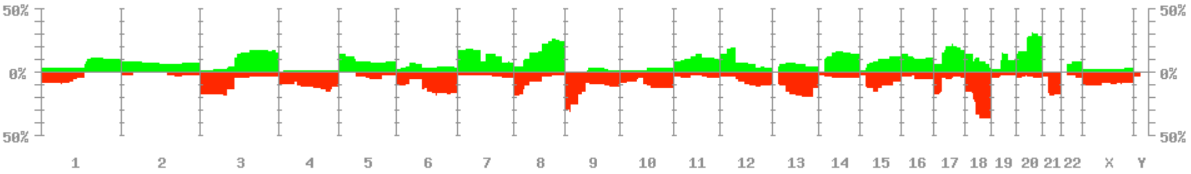

Cholangio-carcinomas (intra- and extrahepatic; 63 cases)

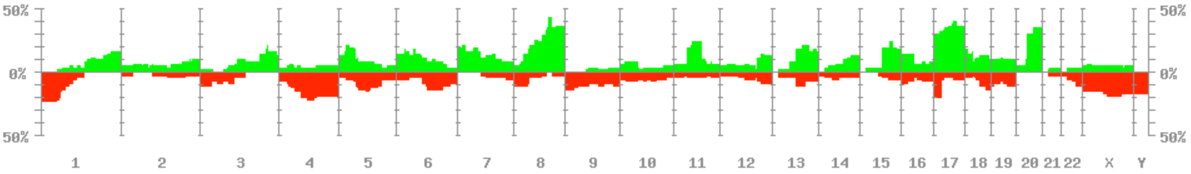

Small-cell lung carcinoma (63 cases)

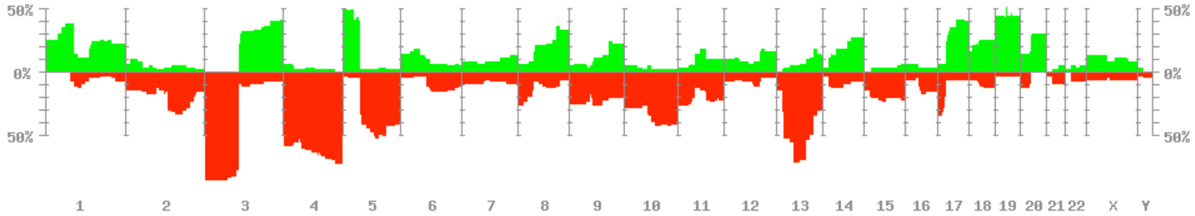

Endometrial carcinoma (56 cases)

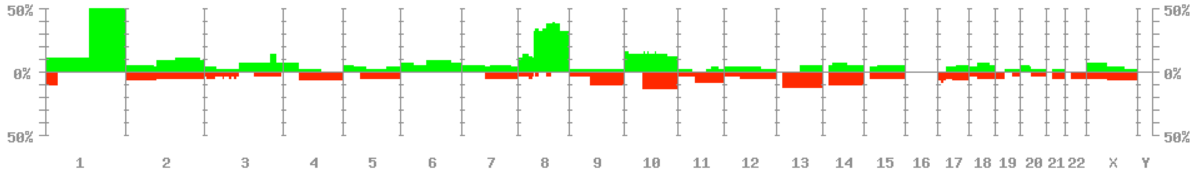

Carcinomas of the vulva (53 cases)

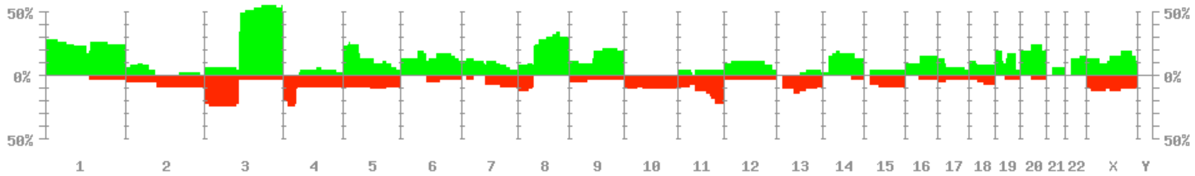

Squamous cell carcinomas of the skin (52 cases)

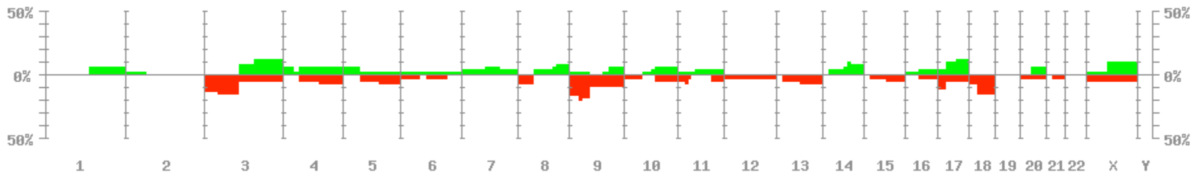

Frequency box plots, only including data from cases with imbalances

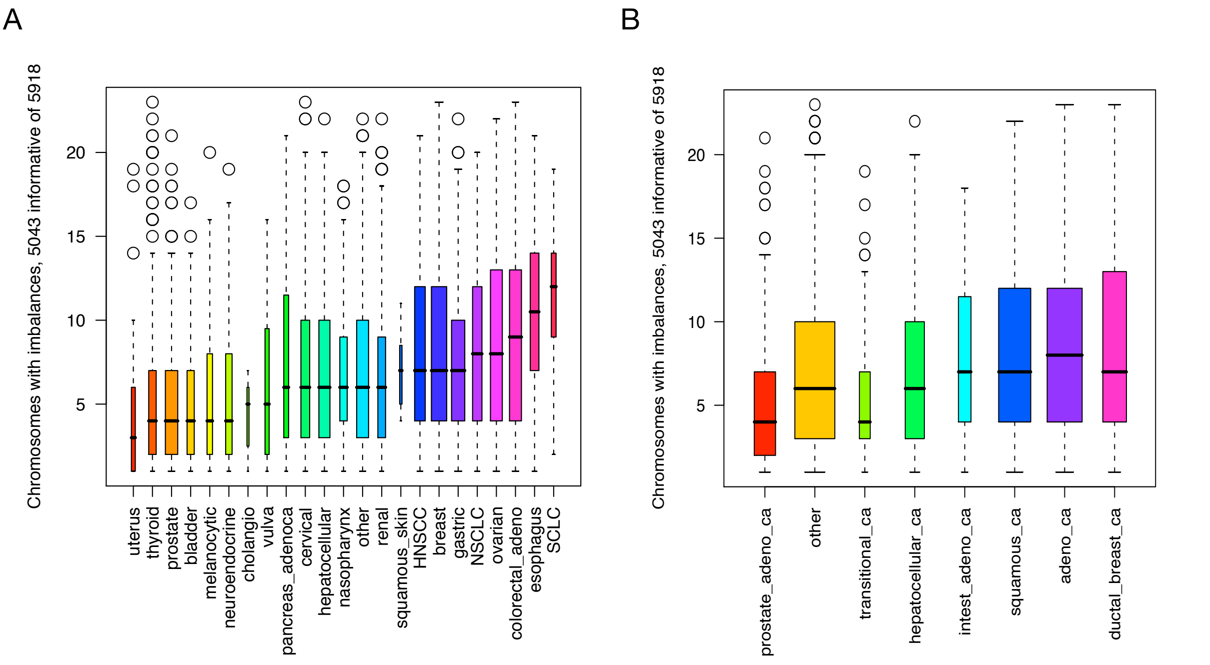

As in figure 1 in the article, these box plots depict the number of imbalances as aberrant chromosomes per case. However, for these plots only cases with imbalances are evaluated. This will correct for some sampling bias, e.g. in entities were many early stage biopsies are included. Figure A) shows the number of aberrations by clinico-pathological entity, figure B) shows the aberrations by histological group.

Additionally, the colors correspond to the coding colors on top of the heatmap in figure 3.
